# The effects of injections of warmed bicarbonate-buffered Lidocaine as a painkiller for patients with trauma

**DOI:** 10.5249/jivr.v7i2.523

**Published:** 2015-07

**Authors:** Reza Azizkhani, Mohsen Forghani, Asieh Maghami-Mehr, Babak Masomi

**Affiliations:** ^*a*^Assistant Professor of Emergency Medicine, Medical School, Isfahan University of Medical Sciences, Isfahan, Iran.; ^*b*^Resident of Emergency Medicine, Medical School, Isfahan University of Medical Sciences, Isfahan, Iran.; ^*c*^Master of Statistics, Department of Statistics, Payam Noor University, Shiraz, Iran.

The importance of pain relief for optimal patient satisfaction at emergency departments has been repeatedly emphasized; a pain-minimizing method with little anesthesia and short duration is crucial. Lidocaine provides excellent local anesthesia for wound repair and is used quite commonly for outpatient surgery when referring cases to the emergency department.^1-7^ Two main methods including warmed Lidocaine and bicarbonate-buffered Lidocaine^1-7^ have been reported to reduce the pain of injection significantly. However, there is no study which has investigated the concurrent effects of warming and adding bicarbonate. Therefore, we investigated pain scores during the injection of warmed lidocaine with bicarbonate during injection for wound management in patients with trauma. A prospective double-blinded clinical trial was designed. 132 patients (27 women), with trauma referring to the emergency department of Al-Zahra Hospital of Isfahan eligible for local anesthesia were recruited. Patients were randomly assigned into three groups of 44 labeled as A, B and C to receive warmed Lidocaine (37° Celsius), bicarbonate-buffered Lidocaine (7.5% Sodium bicarbonate combined with 1% lidocaine with a ratio of 1 to 9 ) and warmed bicarbonate-buffered Lidocaine were used for group A, B and C, respectively. Patients were asked to grade the degree to which they experienced pain during the injection of local anesthesia using a 0 to 10 visual analog scale (VAS). Results of our study showed that there were no significant differences between the three groups with regard to the degree of experienced pain (VAS) during the injection of local anesthesia (A = 1.8±2.8; B = 1.8±3.2; C = 1.6±3.6; p = 0.12). 

In all three groups there were no significant differences between males and females in terms of experienced pain (VAS) (p >0.05). The results of the present study indicate that the degree to which patients experienced pain during the injection of warmed bicarbonate-buffered lidocaine was not significantly different compared to warmed lidocaine and bicarbonate-buffered lidocaine. This implies the necessity to investigate the other factors that may affect pain relief during the injection of local anesthesia for wound repair.
